# Anti-CD4 monoclonal antibody prevents chronic graft-versus-host disease in mice by inducing immune tolerance of CD8^+^ T cells and alleviating thymus injury

**DOI:** 10.3389/fimmu.2024.1460687

**Published:** 2024-12-24

**Authors:** Ziwei Wang, Ruiqi Li, Weijia Fu, Hui Cheng, Yan Zhang, Gusheng Tang, Jianmin Yang, Jianmin Wang, Xiong Ni

**Affiliations:** Department of Hematology, Changhai Hospital, The Second Military Medical University, Shanghai, China

**Keywords:** chronic graft-versus-host disease, CD4 + T cells, anti-CD4 monoclonal antibody, thymic recovery, immune tolerance

## Abstract

**Background:**

Chronic graft-versus-host disease (cGVHD) manifests with characteristics of autoimmune disease with organs attacked by pathogenic helper T cells. Recent studies have highlighted the role of T cells in cGVHD pathogenesis. Due to limited understanding of underlying mechanisms, preventing cGVHD after allogenic hematopoietic cell transplantation (HCT) has become a major challenge.

**Materials and methods:**

Here, we used a representative cGVHD model with the donor C57BL/6 to recipient BALB/c combination. Post-HCT, mice were treated with IgG or anti-CD4 monoclonal antibody. The severity of cGVHD was assessed by evaluating symptoms of cGVHD and histopathology examination (H&E) of target organs. Thymus gland damage and defects of the negative selection were assessed by analyzing the CD4^+^CD8^+^ double-positive thymocytes, cortical thymic epithelial cells and medullary thymic epithelial cells (mTECs). Immunotolerance of CD8^+^ T cells was assessed by detecting the expression of CD80, PD-1, GRAIL and IL-7Rα. Long-term cellular and humoral immunity associated with graft-versus-leukemia (GVL) effects were evaluated through detecting the percentage of CD4^+^ T cells, IgG, IgM and IgA concentrations, and performing tumor challenge experiment.

**Results:**

Donor CD8^+^ T cells caused thymic epithelial cells damage and impaired negative selection in recipients, leading to generation of autoreactive T cells and causing cGVHD. Anti-CD4 mAb treatment promoted immune incompetence of thymus-infiltrating CD8^+^ T cells, facilitated recovery of CD4^+^CD8^+^ thymocytes and regeneration mTECs, and preserved negative-selection, but had no effects on the long-term cellular immunity and humoral immunity, resulting in preservation of GVL effect.

**Conclusion:**

Our results indicate that anti-CD4 mAb therapy early post-HCT allows thymus recovery by inducing the immune tolerance of thymus infiltrated CD8^+^ T cells, thereby alleviating thymic epithelial cells damage, preserving negative selection, and preserving long-term GVL effect at the same time.

## 1 Introduction

Chronic graft-versus-host disease (cGVHD) is a multisystem disease involving almost all tissues and organs, with diverse clinical manifestations similar to those of autoimmune and other immune diseases, such as scleroderma, primary biliary cirrhosis, wasting disease, and chronic immune deficiency syndrome (1). Corticosteroids are currently the basic therapy for the treatment of cGVHD, and a number of clinical trials have investigated the prospective utility of novel drugs to reduce the risk of long-term steroid exposure (2). Over the past decades, the understanding of pathogenesis and underlying biology of cGVHD has advanced rapidly. In cGVHD, donor T cells drive immune pathology through both innate and adaptive immune mechanisms mediated by complement pathways (3, 4). Indeed, allogeneic reactive T cells are key contributors to the development of cGVHD (5).

Notably, extrafollicular CD4^+^ T cells have been shown to be significantly increased in the peripheral blood of cGVHD patients compared to non-cGVHD patients (6). Zeng et al. further demonstrated that the expansion of peripheral T-helper cells within the blood CD4^+^ T cells correlates with the cGVHD severity (7). Therefore, we attempted to investigate the role and mechanism of CD4^+^ T cells clearance using anti-CD4 monoclonal antibody after HCT in attenuating cGVHD in a C57BL/6→BALB/c cGVHD mice model.

Here, we found that early removal of CD4^+^ T cells after HCT could promote immune tolerance of thymus-infiltrating CD8^+^ T cells, attenuate thymic epithelial cell injury, and preserve the GVL effect of HCT in the long term.

## 2 Materials and methods

### 2.1 Mice

Male C57BL/6 (H2b) and BALB/c (H2d) mice, aged 12 to 14 weeks, were utilized as donors and recipients, respectively, to establish the cGVHD model. These animals were procured from Weitong Lihua Experimental Animal Technology (Beijing, China), and maintained in the specific-pathogen-free (SPF) Animal Facility (Changhai Hospital shanghai). All animal experiments were conducted following the institutional animal ethics guidelines and were approved by the Animal care and Use Committee.

### 2.2 Establish cGVHD mice model

BALB/c recipient mice underwent total body irradiation (TBI) using an [^137^Cs] source at a dose of 850 cGy, administered 8–10 hours prior to hematopoietic cell transplantation (HCT). Following irradiation, T-cell-depleted bone marrow cells (TCD-BM, 2.5 × 10^6^) and spleen cells (1.25 × 10^6^) from C57BL/6 donor mice were injected intravenously. T cells were removed from bone marrow cells using anti-mouse CD90.2 microbeads (Miltenyi Biotec, Germany). Recipients receiving TCD-BM and spleen cells were randomly assigned to either the anti-CD4 group or the IgG group. In the anti-CD4 group, mice were administered anti-mouse CD4 monoclonal antibody via tail vein injection (500 µg/mouse, GK1.5, Bio X Cell) on days 0, 14, and 28 post-HCT, whereas mice in the IgG group were treated with anti-mouse IgG (500 µg/mouse on the same schedule; LTF-2, Bio X Cell). For certain experiments, mice receiving only TCD-BM grafts served as controls (TCD-BM group). Clinical cutaneous GVHD scores were assessed following the method described in our previous study (8).

### 2.3 Histological analysis

For the histological analysis of cGVHD, representative tissue samples, including those from the skin, salivary, liver, lung, small intestine, and colon were collected. The tissue was fixed in 4% formaldehyde and than embedded in paraffin. Tissue slices of 6μm from mice were stained with H&E to examine the cGVHD-induced damage. The degree of inflammatory infiltration was graded as previously described (8, 9).

### 2.4 Histoimmunofluorescent staining

Immunofluorescent staining was performed to identify medullary thymic epithelial cells (mTEC) and cortical thymic epithelial cells (cTEC) using Ulex Europaeus Agglutinin 1 (UEA-I) and anti-cytokeratin 8 as previously reported (10). In brief, paraformaldehyde-fixed tissue sections were processed by deparaffinization, hydration, and antigen retrieval. The sections were then blocked and permeabilized with PBS containing 10% donkey serum and 0.1% Triton X-100 for 1 hour. Primary antibodies, including UEA-I (green fluorescence for mTEC) and anti-cytokeratin 8 (red fluorescence for cTEC), were applied overnight at 4°C. Added the corresponding secondary antibodies (S11223, A21209, Thermo Fisher Scientific) and incubated for 1 hour at 25 °C.

### 2.5 Flow cytometry

Monoclonal antibodies targeting CD4 (RM4-5), CD8a (SK1), PD-1 (RMP1-30), IL-7Rα (A7R34), and CD80 (16-10A1) were sourced from eBioscience. Anti-RNF128: FITC (GRAIL) antibody (ARP43311_T100) was acquired from Aviva Systems Biology Corporation (Beijing, China). Antibodies specific for TCRβ (H57-597) and H-2Kb (AF6-88.5) were sourced from BD Pharmingen, while the antibody for CCR9 (242503) was obtained from R&D. Data generated from flow cytometry were processed and analyzed using FlowJo software.

### 2.6 Measurement of immunoglobulins in serum

Serum IgA, IgM and IgG were tested using ELISA kit from MultiSciences (Lianke) Biotechnology Co., Ltd. Each sample was assayed in triplicate, and results were compared to the standards.

### 2.7 Bioluminescent imaging

Mice were administered an intraperitoneal (i.p.) injection of 5×10^6^ luciferase-expressing BCL1 cells (BCL1/Luc+) suspended in 1 ml of PBS. Tumor progression was monitored *in vivo* using imaging techniques as previously described (11). In brief, mice were anesthetized with isoflurane 10-15 min before imaging, and then 200 μl of D-fluorescein solution (15 mg/ml, Goldbio) was injected into each mouse by intraperitoneal injection. Bioluminescent signals were captured using the IVIS imaging system (Caliper Life Sciences).

### 2.8 Statistical analysis

The data were described as mean ± SEM. Diarrhea, body weight, and cutaneous cGVHD scores were assessed using the rank-sum test, while survival rates across groups were compared with the log-rank test. Statistical analyses were determined using Student’s *t*-test (two groups) or one-way ANOVA with Tukey’s *post hoc* test (three groups).

## 3 Results

### 3.1 Anti-CD4 antibodies prevent the development of cGVHD

**Figure 1:**
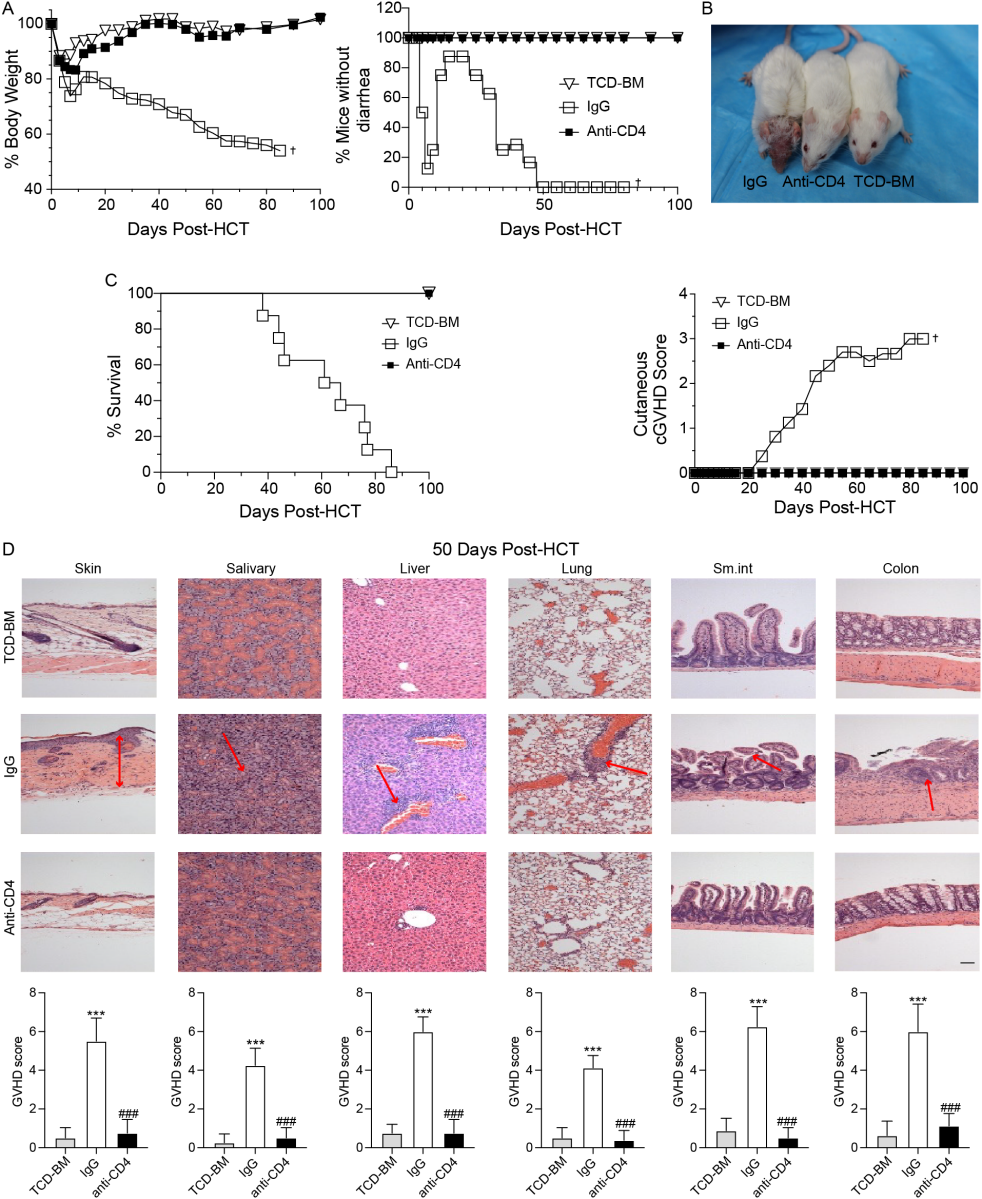
Anti-CD4 antibodies prevent the development of cGVHD. **(A)** Graphs showing percentage of body weight changes (left) and absence of diarrhea (right) in TCD-BM mice (TCD-BM), IgG treated mice (IgG) and Anti-CD4 treated mice (Anti-CD4) after HCT. n=8 per group. **(B)** Representative images of mice (top) at 50-60 days post-HCT and the cutaneous cGVHD score (bottom) from TCD-BM, IgG and Anti-CD4 groups. n=8 per group. **(C)** Graphs showing the survival curves of TCD-BM, IgG and Anti-CD4 groups. n=8 per group. **(D)** Representative H&E -stained sections of skin, salivary, liver, lung, small intestine and colon (top) from TCD-BM, IgG and Anti-CD4 group, with corresponding GVHD scores (mean ± SEM) (bottom). n=8 per group. ^†^indicates death of all recipients in a group. The red arrows indicate representative changes in GVHD targeted tissues. The statistical significance was performed according to one-way ANOVA followed by Tukey’s *post hoc*. ***p < 0.001 versus TCM-BM. ^###^p < 0.001 versus IgG. Scale bar: 100 μm.

As illustrated in **Figure 1A**, mice received TCD-BM alone did not develop cGVHD. In contrast, mice treated with IgG experienced cGVHD symptoms, including diarrhea and weight loss, by day 7 post-HCT. Although these mice initially regained body weight and showed reduced diarrhea around 14 days post-HCT, they exhibited recurrent weight loss and worsening diarrhea starting around day 20, ultimately leading to 100% mortality by approximately day 85 post-HCT (**Figures 1A–C**). Conversely, mice receiving anti-CD4 antibodies treatment steadily recovered body weight without signs of diarrhea, and all mice survived 100 days post-HCT, which was similar to the mice of TCD-BM group (**Figures 1A–C**).

Histopathological analyses of the skin, salivary glands, liver, lung, small intestine, and colon were conducted between 50–60 days after HCT. GVHD pathology scores showed that IgG-treated mice had significantly higher scores in all tissues compared to the TCD-BM and anti-CD4 groups (**Figure 1D**). These results indicated that clearance of donor CD4^+^ T cells effectively attenuated tissue damage caused by cGVHD and improved overall outcomes.

### 3.2 Depletion of donor CD4^+^ T cells promotes recovery of CD4^+^CD8^+^ thymocytes and medullary thymic epithelial cells

**Figure 2:**
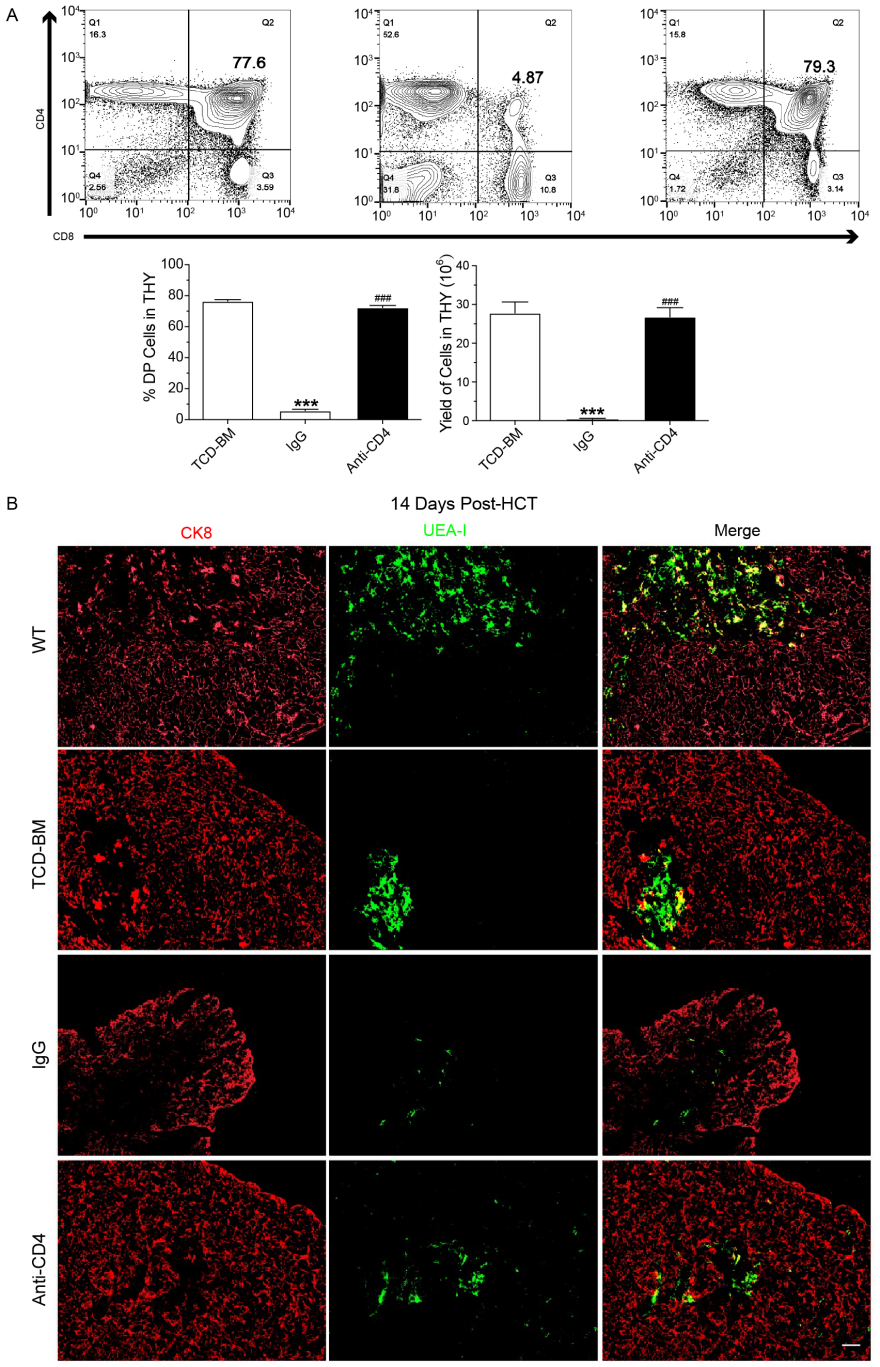
Depletion of donor CD4+ T cells promotes recovery of CD4+CD8+ thymocytes and medullary thymic epithelial cells. **(A)** Representative FACS images (top) at day 60 post-HCT and mean ± SEM of percentage and yield (bottom) of CD8^+^CD4^+^ cells in the thymus of TCD-BM, IgG and Anti-CD4 groups. n=8 per group. **(B)** Representative immunostaining images of CK8 and UEA-I in the thymus of wild type mice (WT) TCD-BM, IgG and Anti-CD4 groups at day14 post-HCT. n=4 per group. Scale bar: 50 μm. The statistical significance was performed according to one-way ANOVA followed by Tukey’s *post hoc*. ***p < 0.001 versus TCM-BM. ^###^p < 0.001 versus IgG.

We then assessed the yield and percentage of CD4^+^CD8^+^ double-positive (DP) thymocytes in TCD-BM group, IgG-treated group, and anti-CD4-treated group on day 60 post-HCT. Mice in the IgG group showed severe lymphopenia, with a significantly reduced number of CD4^+^CD8^+^ DP thymocytes. At day 60 post-HCT the yield and percentage of DP thymocytes in anti-CD4 group were similar to the TCD-BM group (**Figure 2A**).

The thymic medulla, where negative selection occurs during T cell development, relies heavily on the proper function of mTECs. Impaired or incomplete recovery of mTECs after transplantation disrupts negative selection, leading to the generation of autoreactive CD4^+^ T cells and subsequent triggered cGVHD. To examine the effect of anti-CD4 on mTEC recovery, thymic tissues were collected at day 14 post-HCT. And the amount of mTECs and cortical thymic epithelial cells (cTECs) were assessed from each group using anti-cytokeratin 8 (red, cTEC) and UEA-I (green, mTEC) immunofluorescence staining. The results showed a decrease of mTECs after HCT with different severity in TCD-BM, anti-CD4 and IgG groups compared with wide type (WT) BALB/c mice. Three injections of anti-CD4 partially restored the level of mTEC compared with IgG treated mice (**Figure 2B**).

### 3.3 Early depletion of donor CD4^+^ T cells post-transplantation enhances immune dysfunction in thymus infiltrating CD8^+^ T cells

**Figure 3:**
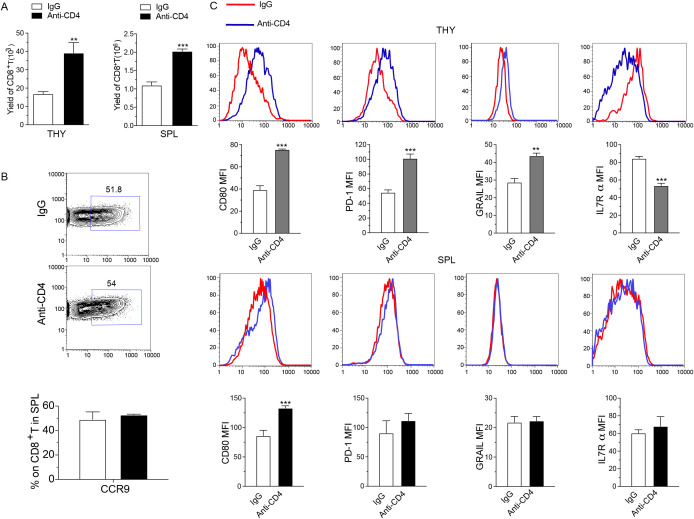
Early depletion of donor CD4+ T cells post-transplantation enhances immune dysfunction in thymus infiltrating CD8+ T cells. **(A)** Yield of donor-derived CD8^+^ T cell in the thymus (left) and spleen (right) from IgG and Anti-CD4 groups at day 7 post-HCT. n=4 per group. **(B)** Representative FACS images (top) and percentage of CCR9 expression on CD8^+^ T cells in the spleen (mean ± SEM) (down) of IgG and Anti-CD4 groups collected at day 7 post-HCT. n=4 per group. **(C)** Representative FACS image and expression levels (mean ± SEM) of CD80, PD-1, GRAIL and IL-7Rα on thymus (THY) and spleen (SPL) infiltrated H2Kb^+^TCRβ^+^CD8^+^ T cells in IgG and anti-CD4 groups collected at day 7 post-HCT. n=4 per group. The statistical significance is performed according to Student’s t-tests (unpaired two-tailed). **p < 0.01 and ***p < 0.001 versus IgG.

At day 7 following HCT, we collected the thymus and spleen tissues, and performed FACS analysis. The results showed that donor-derived CD8^+^ T cells in the thymus and spleen were significantly increased in the anti-CD4 group compared with the IgG group (**Figure 3A**). As CCR9 receptor (CCL25) is predominantly expressed on thymic epithelial cells, we examined the expression level of CCR9 on CD8^+^ T cells isolated from spleens and showed that anti-CD4 therapy did not affect the expression of CCR9 compared to IgG treatment (**Figure 3B**). These suggested that early administration of anti-CD4 monoclonal antibody after transplantation resulted in a greater abundance of donor-derived CD8^+^ T cells in both thymus and spleen.

In order to explore the mechanism of thymus recovery through the higher yield of donor derived CD8^+^ T cells, we analyzed the expression of CD80, PD-1, GRAIL, and IL-7Rα on H-2Kb^+^TCRβ^+^CD8^+^ T cells collected from the thymus and spleen. Results showed that in thymus-infiltrating CD8^+^ T cells, anti-CD4 group displayed elevated levels of CD80, PD-1, and GRAIL and reduced level of IL-7Rα compared to the IgG group (**Figure 3C**). In contrast, anti-CD4 treatment did not affect the expression levels of PD-1, GRAIL, and IL-7Rα on CD8^+^ T cells in the spleen compared with IgG treatment (**Figure 3C**). These results suggested that depletion of CD4^+^ T cell upregulated CD80, PD-1, and GRAIL expression in thymus-infiltrating CD8^+^ T cells while reduced IL-7Rα expression, potentially promoting apoptosis, anergic and exhaustion of CD8^+^ T cells.

### 3.4 Temporary depletion of CD4^+^ T cells *in vivo* does not impair long-term cellular and humoral immunity and the GVL effect

**Figure 4:**
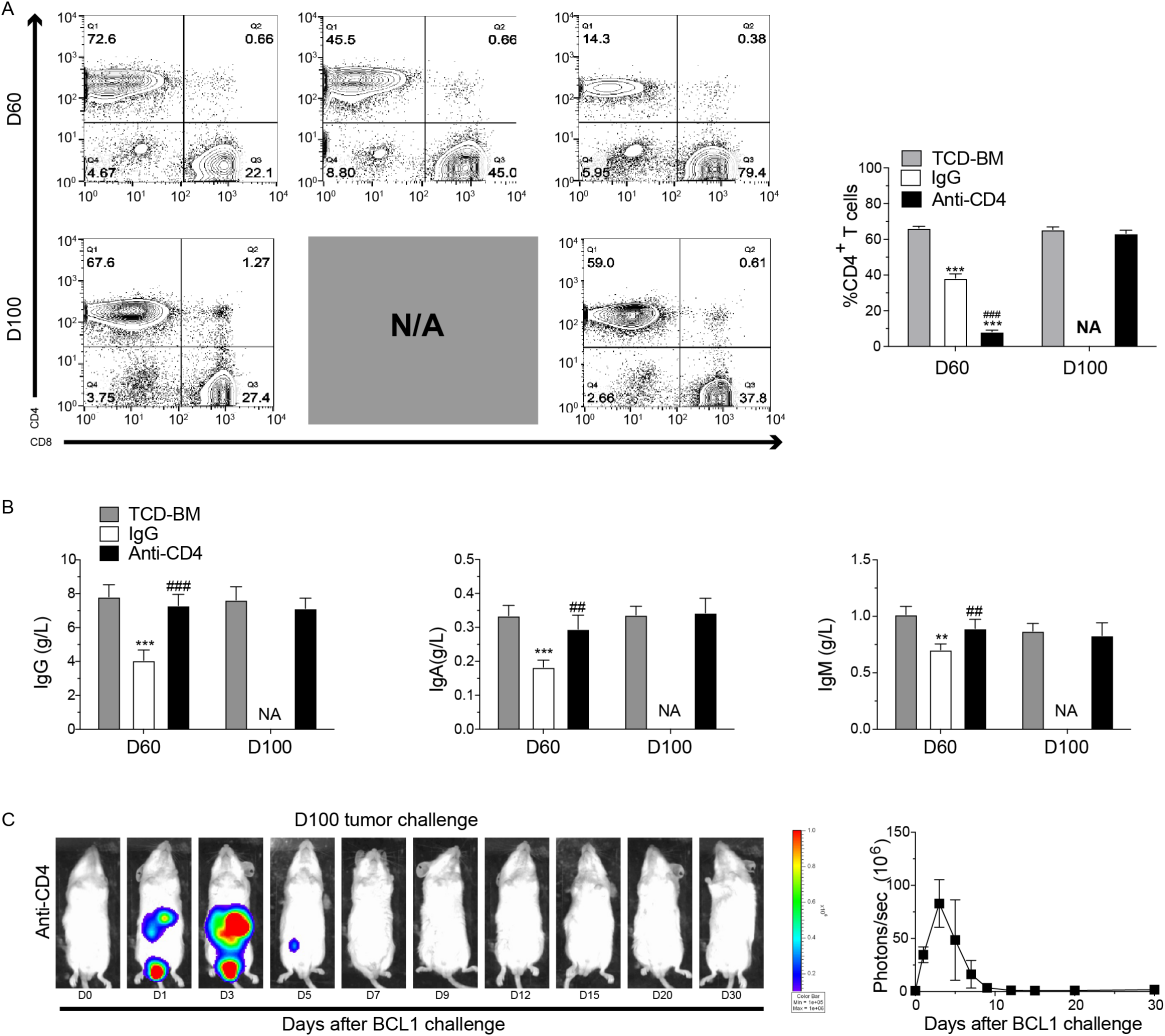
Temporary depletion of CD4+ T cells *in vivo* does not impair long-term cellular and humoral immunity and the GVL effect. **(A)** Representative FACS images (left) and percentage (mean ± SEM) (right) of H2Kb^+^TCRβ^+^CD4^+^ T cells in the spleen from TCD-BM, IgG and Anti-CD4 groups at day 60 and day 100 after HCT. n=4 for each group. **(B)** ELISA results of the concentrations (mean ± SEM) of IgG (left), IgA (middle), and IgM (right) from serum of TCD-BM, IgG and Anti-CD4 groups on day 60 (n=4 per group) and day100 (n=4 per group) after HCT. **(C)** Representative image of *in vivo* bioluminescent imaging (BLI) and the statistic result from each time point after leukemia/lymphoma challenge. n=3 for each group. The statistical significance was performed according to one-way ANOVA followed by Tukey’s *post hoc*. **p < 0.01 and ***p < 0.001 versus TCM-BM. ^##^p < 0.01 and ^###^p < 0.001 versus IgG. NA, Not Applicable.

At 60 and 100 days post-HCT, we collected the spleens and blood samples from each group. Flow cytometry was performed to evaluate percentage of CD4^+^ T cells, while ELISA was used to measure immunoglobulin levels. Results showed a decreased percentage of CD4^+^ T cells in the spleen of the IgG-treated group at both 60 and 100 days post-HCT compared with TCD-BM group (**Figure 4A**). In anti-CD4 group, CD4^+^ T cell levels were also noticeably lower compared to the TCD-BM group at day 60, but this difference was no longer significant by day 100 (**Figure 4A**). Similarly, levels of IgG, IgA, and IgM in the anti-CD4 group were comparable to those in the TCD-BM group at day 100 (**Figure 4B**). These findings indicated that early removing of donor CD4^+^ T cells did not compromise long-term cellular or humoral immunity of HCT.

To evaluate the GVL effect of depletion of CD4^+^ T cells, anti-CD4 group mice were injected with 5×10^6^ BCL1 cells expressing luciferase (BCL1/Luc^+^) 100 days post-HCT. Tumor progression was monitored using bioluminescence imaging (BLI). Mice in the anti-CD4 group eliminated leukemia cells within 7 days after injection, with BLI revealing tumor reduction beginning on day 3 and complete disappearance by day 7 (**Figure 4C**). These findings suggested that temporary depletion of CD4^+^ T cells early post-HCT did not affect the long-term GVL effect.

## 4 Discussion

In this study, we utilized a C57BL/6 (H2b) donor to BALB/c (H2d) recipient mice model of cGVHD to investigate the role and mechanism of depletion of CD4^+^ T cell in preventing cGVHD and protecting thymic function. We hypothesized that transient treatment with anti-CD4 mAb supports reconstruction of CD4^+^CD8^+^ DP thymocytes, partial recovery of mTECs, and induction of immune tolerance in thymus-infiltrating CD8^+^ T cells, thereby mitigating cGVHD. Moreover, our findings confirmed that anti-CD4 mAb treatment does not impair long-term humoral or cellular immunity and preserves the GVL effect. These results provided insights into the potential mechanisms underlying cGVHD and highlight CD4^+^ T cells as a promising therapeutic target for this condition.

Donor CD4^+^ T cells caused significant damage to mTECs, which are critical for the negative selection of T cells, implicating impaired negative selection as a contributing factor to cGVHD pathogenesis (12). Previous study has shown that mTEC damage could initiate the production of autoreactive CD4^+^ T cells, leading to cGVHD (13). In our study, a marked reduction in CD4^+^CD8^+^ thymocytes was observed in cGVHD mice by days 50-60 post-HCT, accompanied by chronic tissue damage of skin, salivary glands, lungs, small intestine, and colon. Treatment with anti-CD4 mAb not only alleviated clinical symptoms, such as weight loss and diarrhea, but also significantly enhanced overall survival. Additionally, anti-CD4 mAb therapy partially restored mTEC numbers, further supporting its therapeutic potential.

PD-L1 could interact with PD-1 and CD80, while T cell activation induces T cells to express PD-1 (14, 15). Peripheral T cell tolerance requires interactions between PD-L1 with both PD-1 and CD80 expressed in naive T cells. Disruption of either interaction can prevent tolerance development and trigger autoimmune responses (16, 17). Our previous study has shown that PD-L1/CD80 interaction enhances the proliferation of alloreactive CD4^+^ conventional T cells (Tcon), which in turn increases Tcon apoptosis (18). Additionally, the E3 ubiquitin ligase GRAIL plays a pivotal role in maintaining peripheral tolerance (19). GRAIL expression increases during induction of T cell anergy, and its absence results in impaired anergy induction (20, 21). Similarly, IL-7 signals are crucial for both T cell and B cell development (22, 23). Its receptor (IL-7R) comprises two subunits: the γ-chain and the α-chain. While T cells express IL-7R in their naive and memory states, they rapidly lose IL-7Rα expression upon activation to maintain immune homeostasis (24). Based on these insights, we hypothesized that depletion of donor CD4^+^ T cells early post-HCT could upregulate the expression of PD-1, CD80, and GRAIL, while downregulate IL-7Rα, which consistent with the characteristics of apoptosis and anergy/exhaustion observed in CD8^+^ T cells.

Our previous research demonstrated that anti-CD4 therapy alleviated acute GVHD by increasing PD-L1 expression, which induced T cell apoptosis and exhaustion via PD-L1/PD-1 interactions (25). Furthermore, we observed that anti-CD4 treatment facilitated the repair of thymic epithelial cells, thereby alleviating cGVHD symptoms. The current study builds upon these results, elucidating how anti-CD4 therapy enhances thymic epithelial cell regeneration and reinstates negative selection. Specifically, our data indicated that anti-CD4 therapy fosters immune tolerance in thymus-infiltrating donor CD8^+^ T cells, reducing their damaging effects on the thymus. Importantly, we also demonstrated that this treatment does not compromise long-term cellular or humoral immunity. Collectively, these findings provided insight into the mechanisms by which anti-CD4 treatment alleviates both acute and chronic GVHD, offering a robust theoretical foundation for its clinical application.

**Figure 5:**
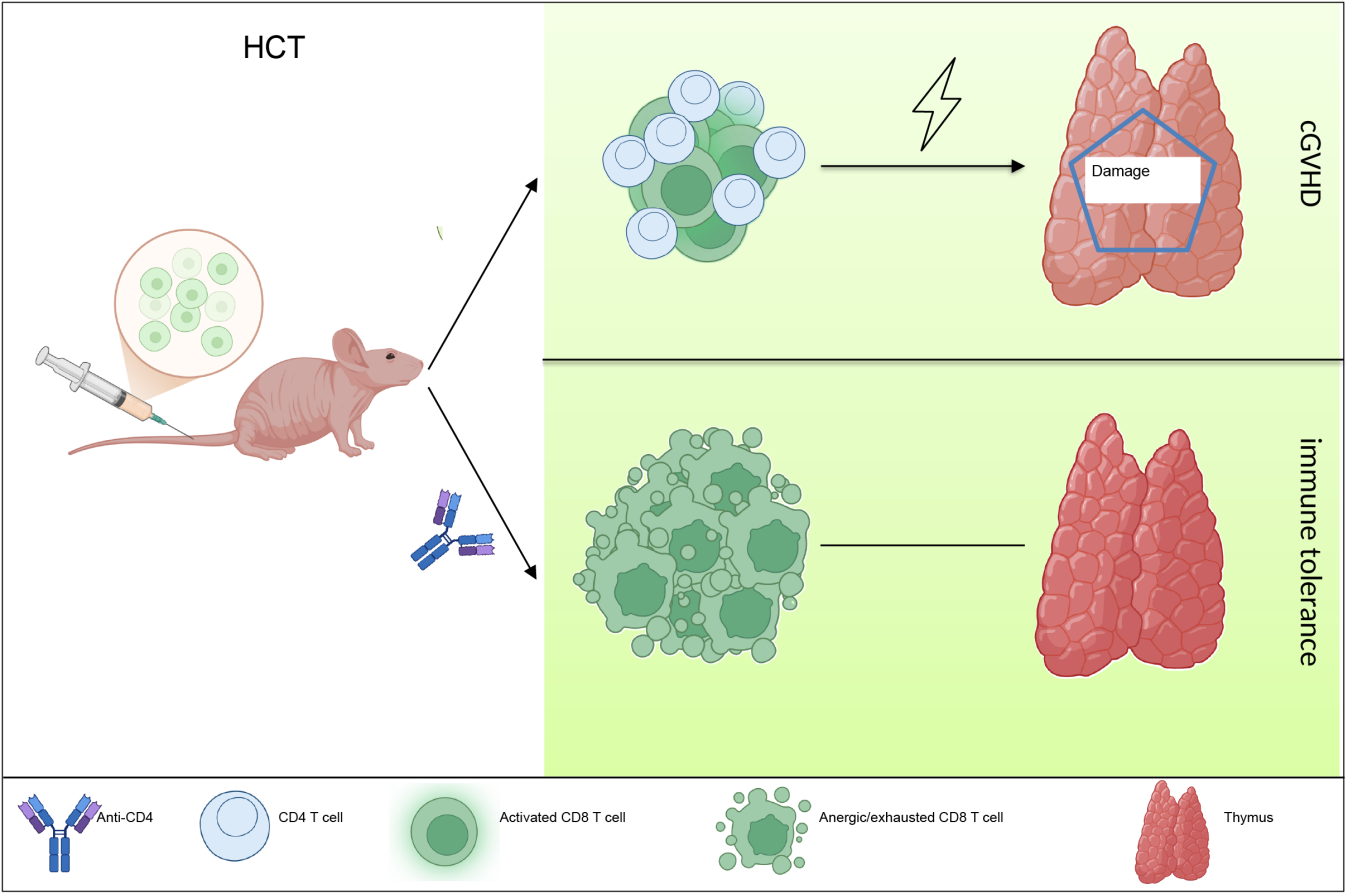
Model of role and mechanism of anti-CD4 in preventing cGVHD.

In summary, our study showed that temporary anti-CD4 mAb treatment early post transplantation induced immune tolerance of donor-derived CD8^+^ T cells in the thymus, thereby allowed regeneration of mTECs, recovery of thymic negative selection and prevent cGVHD in the end (**Figure 5**). These findings have important clinical potential.

## Data Availability

The original contributions presented in the study are included in the article/supplementary material. Further inquiries can be directed to the corresponding author.

## References

[1] Zeiser R Blazar BR. Pathophysiology of chronic graft-versus-host disease and therapeutic targets. N Engl J Med. (2017) 377:2565–79. doi: 10.1056/NEJMra1703472 29281578

[2] Gonzalez RM Pidala J. Evolving therapeutic options for chronic graft-versus-host disease. Pharmacotherapy. (2020) 40:756–72. doi: 10.1002/phar.2427 32441379

[3] Flynn R Du J Veenstra RG Reichenbach DK Panoskaltsis-Mortari A Taylor PA. Increased T follicular helper cells and germinal center B cells are required for cgvhd and bronchiolitis obliterans. Blood. (2014) 123:3988–98. doi: 10.1182/blood-2014-03-562231 PMC4064335 24820310

[4] Ricklin D Hajishengallis G Yang K Lambris JD. Complement: A key system for immune surveillance and homeostasis. Nat Immunol. (2010) 11:785–97. doi: 10.1038/ni.1923 PMC2924908 20720586

[5] Appelbaum FR. Haematopoietic cell transplantation as immunotherapy. Nature. (2001) 411:385–9. doi: 10.1038/35077251 11357147

[6] Jin H Yang KB Zhang HY Chen YQ Qi HZ Fan ZP. Expansion of circulating extrafollicular helper T-like cells in patients with chronic graft-versus-host disease. J Autoimmun. (2019) 100:95–104. doi: 10.1016/j.jaut.2019.03.006 30878167

[7] Kong X Wu X Wang B Zeng D Cassady K Nasri U. Trafficking between clonally related peripheral T-helper cells and tissue-resident T-helper cells in chronic Gvhd. Blood. (2022) 140:2740–53. doi: 10.1182/blood.2022016581 PMC9935547 36084473

[8] Young JS Wu T Chen Y Zhao D Liu H Yi T. Donor B cells in transplants augment clonal expansion and survival of pathogenic Cd4+ T cells that mediate autoimmune-like chronic graft-versus-host disease. J Immunol. (2012) 189:222–33. doi: 10.4049/jimmunol.1200677 PMC3746987 22649197

[9] Zhao D Young JS Chen YH Shen E Yi T Todorov I. Alloimmune response results in expansion of autoreactive donor Cd4+ T cells in transplants that can mediate chronic graft-versus-host disease. J Immunol. (2011) 186:856–68. doi: 10.4049/jimmunol.1002195 PMC3891909 21149609

[10] Jin H Ni X Deng RS Song QX Young J Cassady K. Antibodies from donor B cells perpetuate cutaneous chronic graft-versus-host disease in mice. Blood. (2016) 127:2249–60. doi: 10.1182/blood-2015-09-668145 PMC4859199 26884373

[11] Na IK Lu SX Yim NL Goldberg GL Tsai J Rao U. The cytolytic molecules fas ligand and trail are required for murine thymic graft-versus-host disease. J Clin Invest. (2010) 120:343–56. doi: 10.1172/Jci39395 PMC2798682 19955659

[12] Muller AMS Min D Wernig G Levy RB Perez VL Herretes S. Modeling chronic graft-versus-host disease in Mhc-matched mouse strains: genetics, graft composition, and tissue targets. Biol Blood Marrow Transplant. (2019) 25:2338–49. doi: 10.1016/j.bbmt.2019.08.001 31415899

[13] Wu T Young JS Johnston H Ni X Deng RS Racine J. Thymic damage, impaired negative selection, and development of chronic graft-versus-host disease caused by donor Cd4(+) and Cd8(+) T cells. J Immunol. (2013) 191:488–99. doi: 10.4049/jimmunol.1300657 PMC3746979 23709681

[14] Keir ME Butte MJ Freeman GJ Sharpel AH. Pd-1 and its ligands in tolerance and immunity. Annu Rev Immunol. (2008) 26:677–704. doi: 10.1146/annurev.immunol.26.021607.090331 18173375 PMC10637733

[15] Li XF Deng RS He W Liu C Wang M Young J. Loss of B7-H1 expression by recipient parenchymal cells leads to expansion of infiltrating donor Cd8(+) T cells and persistence of graft-versus-host disease. J Immunol. (2012) 188:724–34. doi: 10.4049/jimmunol.1102630 PMC3891913 22156590

[16] Yi TS Li XF Yao S Wang L Chen YH Zhao DC. Host apcs augment *in vivo* expansion of donor natural regulatory T cells via B7h1/B7.1 in allogeneic recipients. J Immunol. (2011) 186:2739–49. doi: 10.4049/jimmunol.1002939 PMC3786569 21263067

[17] Tsushima F Yao S Shin T Flies A Flies S Xu HY. Interaction between B7-H1 and Pd-1 determines initiation and reversal of T-cell anergy. Blood. (2007) 110:180–5. doi: 10.1182/blood-2006-11-060087 PMC1896111 17289811

[18] Deng RS Cassady K Li XF Yao S Zhang MF Racine J. B7h1/cd80 interaction augments Pd-1-dependent T cell apoptosis and ameliorates graft-versus-host disease. J Immunol. (2015) 194:560–74. doi: 10.4049/jimmunol.1402157 PMC4282988 25488990

[19] Schwartz RH. T cell anergy. Annu Rev Immunol. (2003) 21:305–34. doi: 10.1146/annurev.immunol.21.120601.141110 12471050

[20] Anandasabapathy N Ford GS Bloom D Holness C Paragas V Seroogy C. Grail: an E3 ubiquitin ligase that inhibits cytokine gene transcription is expressed in anergic Cd4(+) T cells. Immunity. (2003) 18:535–47. doi: 10.1016/S1074-7613(03)00084-0 12705856

[21] Kriegel MA Rathinam C Flavell RA. E3 ubiquitin ligase grail controls primary T cell activation and oral tolerance. P Natl Acad Sci USA. (2009) 106:16770–5. doi: 10.1073/pnas.0908957106 PMC2757842 19805371

[22] Mai HL Boeffard F Longis J Danger R Martinet B Haspot F. Il-7 receptor blockade following T cell depletion promotes long-term allograft survival. J Clin Invest. (2014) 124:1723–33. doi: 10.1172/Jci66287 PMC3973119 24569454

[23] Leonard WJ. Cytokines and immunodeficiency diseases. Nat Rev Immunol. (2001) 1:200–8. doi: 10.1038/35105066 11905829

[24] Barata JT Durum SK Seddon B. Flip the coin: Il-7 and Il-7r in health and disease. Nat Immunol. (2019) 20:1584–93. doi: 10.1038/s41590-019-0479-x 31745336

[25] Ni X Song QX Cassady K Deng RS Jin H Zhang MF. Pd-L1 interacts with Cd80 to regulate graft-versus-leukemia activity of donor Cd8(+) T cells. J Clin Invest. (2017) 127:1960–77. doi: 10.1172/Jci91138 PMC5409099 28414296

